# Identification of pediatric septic shock subclasses based on genome-wide expression profiling

**DOI:** 10.1186/1741-7015-7-34

**Published:** 2009-07-22

**Authors:** Hector R Wong, Natalie Cvijanovich, Richard Lin, Geoffrey L Allen, Neal J Thomas, Douglas F Willson, Robert J Freishtat, Nick Anas, Keith Meyer, Paul A Checchia, Marie Monaco, Kelli Odom, Thomas P Shanley

**Affiliations:** 1 Cincinnati Children's Hospital Medical Center and Cincinnati Children's Research Foundation, Department of Pediatrics, University of Cincinnati College of Medicine, Cincinnati, OH, USA; 2 Children's Hospital and Research Center Oakland, Oakland, CA, USA; 3 The Children's Hospital of Philadelphia, Philadelphia, PA, USA; 4 Children's Mercy Hospital, Kansas City, MO, USA; 5 Penn State Children's Hospital, Hershey, PA, USA; 6 University of Virginia, Charlottesville, VA, USA; 7 Children's National Medical Center, Washington, DC, USA; 8 Children's Hospital of Orange County, Orange, CA, USA; 9 Miami Children's Hospital, Miami, FL, USA; 10 St Louis Children's Hospital, St Louis, MO USA; 11 C.S. Mott Children's Hospital at the University of Michigan, Ann Arbor, MI, USA

## Abstract

**Background:**

Septic shock is a heterogeneous syndrome within which probably exist several biological subclasses. Discovery and identification of septic shock subclasses could provide the foundation for the design of more specifically targeted therapies. Herein we tested the hypothesis that pediatric septic shock subclasses can be discovered through genome-wide expression profiling.

**Methods:**

Genome-wide expression profiling was conducted using whole blood-derived RNA from 98 children with septic shock, followed by a series of bioinformatic approaches targeted at subclass discovery and characterization.

**Results:**

Three putative subclasses (subclasses A, B, and C) were initially identified based on an empiric, discovery-oriented expression filter and unsupervised hierarchical clustering. Statistical comparison of the three putative subclasses (analysis of variance, Bonferonni correction, *P *< 0.05) identified 6,934 differentially regulated genes. K-means clustering of these 6,934 genes generated 10 coordinately regulated gene clusters corresponding to multiple signaling and metabolic pathways, all of which were differentially regulated across the three subclasses. Leave one out cross-validation procedures indentified 100 genes having the strongest predictive values for subclass identification. Forty-four of these 100 genes corresponded to signaling pathways relevant to the adaptive immune system and glucocorticoid receptor signaling, the majority of which were repressed in subclass A patients. Subclass A patients were also characterized by repression of genes corresponding to zinc-related biology. Phenotypic analyses revealed that subclass A patients were younger, had a higher illness severity, and a higher mortality rate than patients in subclasses B and C.

**Conclusion:**

Genome-wide expression profiling can identify pediatric septic shock subclasses having clinically relevant phenotypes.

## Background

While septic shock is fundamentally an infection-based disease entity, it is not a singular, homogenous disease in the traditional sense. Rather, septic shock is more akin to a syndrome or a broad, heterogeneous disease classification within which likely exist several disease subclasses. The concept of septic shock subclasses is clinically relevant in that potentially it could have major implications for the design of more specifically targeted therapies [1].

Physiology-based subclassifications of septic shock are well recognized and have clear implications for hemodynamic management [2-4]. More recently, there have been attempts to biologically subclassify patients with septic shock using blood-derived biomarkers. For example, a previous clinical trial centered on an anti-tumor necrosis factor antibody strategy used serum interleukin-6 concentrations to identify and stratify septic shock patients with a higher severity of illness, ostensibly to select a patient population that could potentially derive a greater benefit from immune modulation therapy [5,6]. We recently identified a subclass of children with septic shock having a high probability of survival with standard care based on admission serum interleukin-8 levels [7]. While the ease of this type of patient subclassification is clinically appealing, it is overly simplistic from a biological standpoint given the complexity and heterogeneity of septic shock [1].

A potentially more comprehensive approach to subclassification of septic shock involves genome-wide expression profiling based on microarray technology and bioinformatics [8]. Our previous genome-wide expression studies demonstrated and validated that pediatric septic shock is characterized by early, persistent, and concomitant repression of gene programs corresponding to the adaptive immune system and to zinc-related biology [9-13]. Herein we tested the hypothesis that pediatric septic shock subclasses can be discovered through genome-wide expression profiling.

## Methods

### Patients

The study protocol was approved by the individual Institutional Review Boards of each participating institution (*N *= 11 institutions). Children ≤10 years of age admitted to the pediatric intensive care unit and meeting published, pediatric-specific criteria for septic shock were eligible for the study [14]. Controls were recruited from the ambulatory departments of participating institutions using the following exclusion criteria: a recent febrile illness (within 2 weeks), recent use of anti-inflammatory medications (within 2 weeks), or any history of chronic or acute disease even remotely associated with inflammation. The median age (intra-quartile range (IQR)) for the control cohort (*N *= 32) was 1.6 years (0.2 to 3.7 years). There were 19 males and 13 females in the control cohort.

### Sample and data collection

After obtaining informed consent from parents or legal guardians, blood samples were obtained within 24 hours of initial presentation to the pediatric intensive care unit with septic shock. Severity of illness was calculated using the pediatric risk of mortality (PRISM) III score [15]. Organ failure was defined using pediatric-specific criteria [14,16]. Annotated clinical and laboratory data were collected daily while in the pediatric intensive care unit. Clinical, laboratory, and biological data were entered and stored using a web-based database developed locally.

### RNA extraction and microarray hybridization

The data and protocols described in this manuscript are compliant with the minimum information about a microarray experiment (MIAME) and are deposited in the National Center for Biotechnology Information Gene Expression Omnibus (GEO) under accession number GSE13904 (GEO, ). All of the controls and 67 of the patients with septic shock have been previously reported in analyses addressing completely different questions than that addressed in the current study [9,11-13]. An additional 31 patients in the septic shock cohort have not been previously reported. Total RNA was isolated from whole blood samples using the PaxGene™ blood RNA system (PreAnalytiX, Qiagen/Becton Dickson, Valencia, CA, USA) according the manufacturer's specifications. Microarray hybridization was performed by the Affymetrix Gene Chip Core facility at the Cincinnati Children's Hospital Research Foundation as previously described using the Human Genome U133 Plus 2.0 GeneChip (Affymetrix, Santa Clara, CA, USA) [9,11-13].

### Data analysis

Analyses were performed using one patient sample per chip. Image files were captured using an Affymetrix GeneChip Scanner 3000. CEL files were subsequently preprocessed using robust multiple-array average (RMA) normalization and GeneSpring GX 7.3 software (Agilent Technologies, Palo Alto, CA, USA). All signal intensity-based data were used after RMA normalization, which specifically suppresses all but significant variation among lower intensity probe sets [17]. All chips representing patient samples were then normalized to the respective median values of the controls. Lists of differentially regulated genes were generated using a series of expression and statistical filters embedded in the GeneSpring GX 7.3 software. Further details regarding these filters will be provided in the Results section.

Gene lists of differentially expressed genes were primarily analyzed using the Ingenuity Pathways Analysis (IPA) application (Ingenuity Systems, Redwood City, CA, USA) that provides a tool for discovery of signaling pathways and gene networks within the uploaded gene lists as previously described [12,18]. Adjunct analyses of gene lists were performed using the National Institutes of Health Database for Annotation, Visualization and Integrated Discovery (DAVID) [19]. Both applications are based on the established biomedical literature and use specific approaches to estimate significance (*P *values) based on non-redundant representations of the microarray chip and to convert the uploaded gene lists to gene lists containing a single value per gene. The *P *values provide an estimate of the probability that a given enrichment is present by chance alone and are derived using corrections for multiple comparisons.

## Results

### Initial identification of putative septic shock subclasses

**Figure 1:**
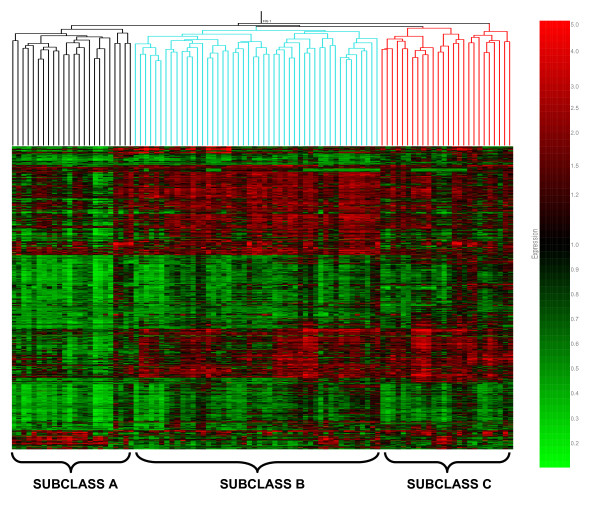
**Unsupervised hierarchical clustering of 98 patients with septic shock (horizontal dimension) and 6,099 genes (vertical dimension) derived from a discovery-oriented filtering approach**. Both the condition tree (patient clustering) and the gene tree are based on the Pearson correlation similarity measurement. The first- and second-order branching patterns of the condition tree were used to identify the putative septic shock classes and are colored for illustrative purposes based on three major putative septic shock subclasses.

The first step toward identification of septic shock subclasses involved derivation of an initial working list of genes differentially regulated between patients with septic shock (*N *= 98) and controls (*N *= 32). The initial working list was derived using an empiric, discovery-oriented expression filter designed to select genes that were increased or decreased ≥2-fold in at least 25%, but not more than 50% of patients with septic shock, relative to the median of controls. This expression filter yielded a working list of 6,099 genes that were subjected to unsupervised hierarchical clustering as shown in Figure 1. An *a priori *decision was made to identify the putative major septic shock subclasses based on the first- and second-order branching patterns of the condition tree (top of Figure 1). Using this strategy, Figure 1 suggested the existence of three major septic subclasses that we arbitrarily designated as subclasses A, B, and C.

### Differential gene expression across septic shock subclasses

To determine if there are significant differences in gene expression across the putative septic shock subclasses, we carried out a statistical test using a three-group analysis of variance (ANOVA), all genes on the microarray (54,641), and the three putative subclasses as the comparison groups. When we applied a Benjamini-Hochberg false discovery rate of 0.1% the resulting gene list consisted of more than 20,000 genes. While this result suggests that the three putative septic shock classes have highly significant differences in gene expression, a working gene list of more than 20,000 genes is excessively large for practical analysis. Accordingly, we applied a more stringent correction for multiple comparisons (Bonferroni; *P *< 0.05) and thus generated a working list of 6,934 genes differentially regulated between the three putative septic shock subclasses.

**Figure 2:**
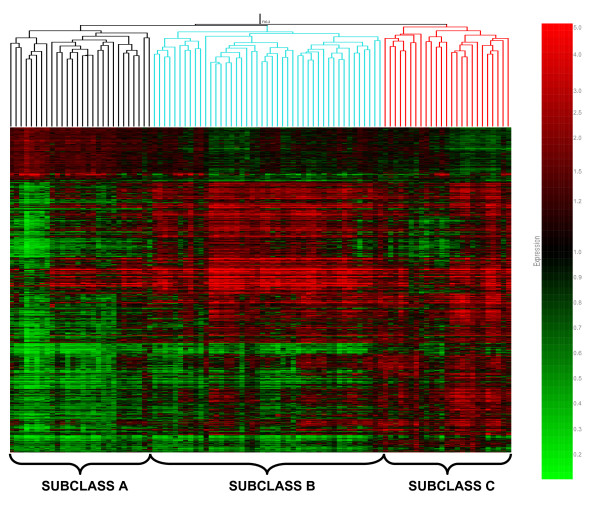
**Unsupervised hierarchical clustering of 98 patients with septic shock (horizontal dimension) and 6,934 genes (vertical dimension) derived from a three group analysis of variance**. Both the condition tree (patient clustering) and the gene tree are based on the Pearson correlation similarity measurement. The first- and second-order branching patterns of the condition tree are colored for illustrative purposes based on septic shock subclasses A, B, and C.

These 6,934 genes were then subjected to unsupervised hierarchical clustering as shown in Figure 2. Based on the first- and second-order branching patterns of the condition tree (top of Figure 2), the differential patterns of gene expression demonstrate the existence of three major subclasses of patients with septic shock (subclasses A, B, and C). Of the patients designated subclass A in Figure 1, 75% belonged to subclass A in Figure 2; 82% of the patients designated subclass B in Figure 1 belonged to subclass B in Figure 2; 80% of the patients designated subclass C in Figure 1 belonged to subclass C in Figure 2.

**Figure 3:**
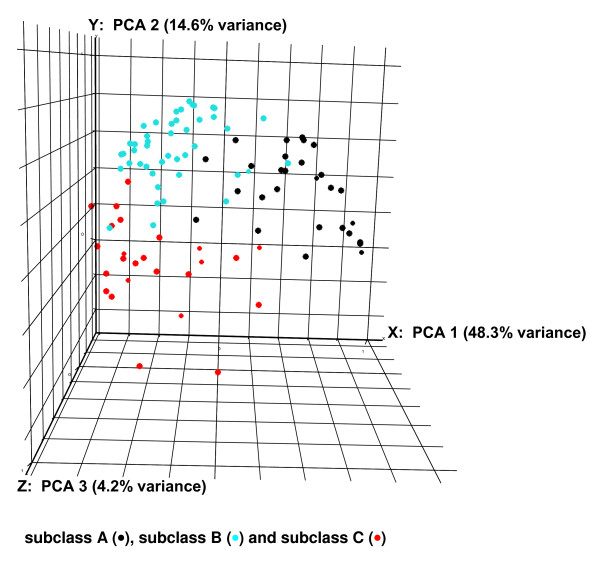
**Three-dimensional principal component analysis (mean centering and scaling) based on the 6,934 genes illustrated in Figure 2**. Individual patients are plotted based on their respective positions along the three axes derived from principal component analysis. Patient subclassifications are indicated by color.

Further evidence for the existence of subclasses A, B, and C was derived by conducting principal component analysis based on the above 6,934 genes and the individual patients in each of the subclasses indentified in Figure 2. Principal component analysis is a mathematical vector space transformation which allows for reduction of multidimensional data sets to lower dimensions (principle components) accounting for variability in the data set [20]. As shown in Figure 3, the principal component analysis based on three dimensions, accounting for 67.1% of the variance in gene expression, provided a high degree of separation between the three septic shock subclasses. Thus, identifiable subclasses of patients with septic shock exist based on genome-level expression patterns.

### Clinical phenotypes of the septic shock subclasses

Table 1 provides the demographic and clinical characteristics of the three septic shock subclasses identified in Figure 2. Patients in subclass A had a significantly higher illness severity level (PRISM III score), a greater degree of organ failure, and a higher mortality rate, compared with patients in subclasses B and C. Patients in subclass A also had a significantly higher incidence of documented Gram-positive bacterial infection, compared with patients in subclass C, and were significantly younger, compared with patients in subclass B. A significantly greater proportion of patients in subclass B received hydrocortisone for cardiovascular shock compared with subclass C. None of the other clinical characteristics listed in Table 1 were significantly different between the three septic subclasses. Thus, the three septic shock classes identified through differential genome-wide expression patterns have significant differences in clinically relevant phenotypes.

**Table 1:** Demographic and clinical data for the septic shock subclasses indentified in Figure 2.

	**Subclass A**	**Subclass B**	**Subclass C**
Number of patients	28	45	25

Median age in years (IQR)	0.3 (0.1–2.7)	4.3 (1.9–7.3)^1^	2.0 (0.8–2.7)

Number of males/females	19/9	19/26	14/11

Number of deaths (%)	10 (36)^2^	5 (11)	3 (12)

Median pediatric risk of mortality (PRISM) score (IQR)	20.5 (12.5–32.5)^2^	15.0 (10.0–21.0)	15.0 (10.7–19.2)

Maximum number of organ failures (IQR)^3^	3 (3–4)^2^	2 (2–3)	2 (2–2)

Number with co-morbidity (%)^4^	10 (36)	20 (44)	11 (44)

Number with immune suppression (%)^5^	7 (25)	14 (31)	2 (8)

Number receiving hydrocortisone (%)^6^	8 (29)	22 (49)^5^	5(20)

Number with Gram-positive bacteria (%)^7^	11 (39)^8^	10 (22)	2 (8)

Number with Gram-negative bacteria (%)	3 (11)	9 (20)	8 (32)

Number with negative cultures (%)	11 (39)	24 (53)	10 (40)

^1^*P *< 0.05 versus subclasses A and C, Mann-Whitney.^2^*P *< 0.05 versus subclasses B and C, Chi-square.^3^Refers to the maximum number of organ failures during the initial 7 days of admission to the pediatric intensive care unit.^4^Refers to patients having any major diagnosis in addition to septic shock (for example, trauma, sickle cell disease, congenital heart disease, liver failure, and so on).^5^Refers to patients with immune deficiency secondary to an intrinsic documented defect of the immune system, or patients receiving immune-suppressive medications (for example, calcineurin inhibitors or high dose steroids).^6^For cardiovascular shock.^7^All bacterial culture data refer to samples obtained from bodily fluids that are normally sterile (that is, blood, urine, cerebral spinal fluid, broncho-alveolar lavage, and/or peritoneal fluid).^8^*P *< 0.05 versus subclass C, Chi-square. IQR = intra-quartile range.

### K-means clustering and pathway analysis

**Figure 4:**
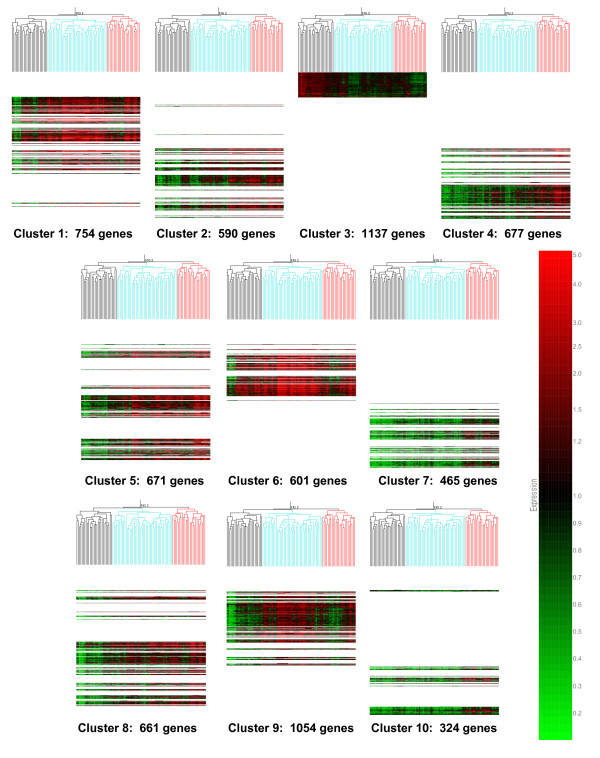
**K-means clustering of 98 patients with septic shock (horizontal dimension) and the 6,934 genes (vertical dimension) shown in Figure 2**. The K-means clustering algorithm is based on 100 iterations, the Pearson correlation similarity measurement, and a maximum return of 10 clusters. The first- and second-order branching patterns of the condition trees are colored for illustrative purposes based on septic shock subclasses A, B, and C.

To derive further biological information from the 6,934 genes depicted in Figure 2, we next sought to identify coordinately regulated gene clusters by conducting K-means clustering based on a maximum return of 10 clusters [21]. Figure 4 illustrates how the K-means clustering algorithm arranged the 6,934 genes into 10 clusters of coordinately regulated genes. All of the 6,934 genes are represented in 1 of the 10 clusters, with no genes unclassified.

The gene lists corresponding to each of the 10 clusters depicted in Figure 4 were individually uploaded to the IPA application and the analytical output was focused on enrichment for genes corresponding to signaling and metabolic pathways. Table 2 provides the top five (based on *P *values) signaling and metabolic pathways represented in each cluster. All clusters were enriched for signaling and metabolic pathways potentially relevant to the pathobiology of septic shock. Thus, the 10 K-means clusters of coordinately regulated genes that define septic shock subclasses A, B, and C, are biologically plausible in that they are broadly enriched for signaling and metabolic pathways relevant to the pathobiology of septic shock.

**Table 2:** Ingenuity Pathways Analysis-derived signaling pathways corresponding to the 10 individual K-means clusters depicted in Figure 4.

	**Pathway**	***P *value**	**Number of genes**
CLUSTER 1			
	Erythropoietin signaling	2.0E-8	14
	B cell receptor signaling	2.6E-8	20
	Leukocyte extravasation signaling	2.8E-8	23
	Triggering receptor expressed on myeloid cells signaling	2.3E-7	12
	Janus kinase (JAK)/signal transducers and activator of transcription (STAT) signaling	2.8E-7	12
CLUSTER 2			
	Interferon signaling	8.7E-5	5
	Erythropoietin signaling	7.4E-4	6
	Insulin receptor signaling	1.0E-3	8
	B cell receptor signaling	2.3E-3	8
	JAK/STAT signaling	2.7E-3	5
CLUSTER 3			
	Axon guidance signaling	1.8E-6	38
	Methane metabolism	2.9E-3	4
	Coagulation system	1.9E-2	5
	Calcium signaling	2.0E-2	14
	Glycine, serine, and threonine metabolism	2.9E-2	7
CLUSTER 4			
	One carbon pool by folate	2.4E-3	3
	Protein ubiquitination pathway	2.4E-3	8
	Interleukin-8 signaling	2.1E-4	6
	Glucocorticoid receptor signaling	4.1E-2	7
	Glycine, serine, and threonine metabolism	5.5E-2	3
CLUSTER 5			
	B cell receptor signaling	1.2E-5	11
	Nuclear factor kappa-light-chain-enhancer of activated B cells (NF-κB) signaling	6.9E-5	10
	Retinoic acid receptor activation	1.2E-3	9
	Peroxisome proliferator-activated receptor (PPARα)/retinoid × receptor (RXRα) activation	1.3E-3	9
	PI3 kinase/Akt signaling	2.0E-3	7
CLUSTER 6			
	p38 mitogen-activated protein kinase (MAPK) signaling	7.3E-5	10
	Toll-like receptor signaling	9.3E-4	6
	PPARα/RXRα activation	2.2E-3	11
	Ephrin receptor signaling	2.2E-3	11
	Sphingolipid metabolism	2.8E-3	6
CLUSTER 7			
	Interleukin-4 signaling	1.4E-2	4
	Antigen presentation pathway	1.5E-2	3
	Glyoxylate and dicarboxylate metabolism	3.3E-2	2
	B cell receptor signaling	5.2E-2	5
	Citrate cycle	6.9E-2	2
CLUSTER 8			
	Epidermal growth factor signaling	8.3E-3	4
	Wnt/β-catenin signaling	9.6E-3	8
	Ceramide signaling	1.3E-2	5
	Epidermal growth factor (ERK)/MAPK signaling	1.6E-2	8
	Huntington's disease signaling	1.8E-2	9
CLUSTER 9			
	Death receptor signaling	1.4E-5	11
	B cell receptor signaling	2.8E-5	17
	Integrin signaling	5.1E-5	20
	Huntington's disease signaling	7.0E-5	21
	EGF signaling	2.1E-4	8
CLUSTER 10			
	T cell receptor signaling	2.7E-7	9
	Natural killer cell signaling	5.4E-5	7
	Chemokine signaling	3.0E-2	3
	NF-κB signaling	4.4E-2	4
	Stress-activated protein kinase/c-Jun NH_2_-terminal kinase signaling	5.3E-2	3

### Leave on out cross-validation and top predictor gene derivation

The above data demonstrate the existence of septic shock subclasses, in very broad terms, based on more than 6,000 differentially regulated genes and 10 K-means clusters consisting of between 300 and 1,100 genes. Herein we sought to refine the gene expression patterns that best distinguish the three septic shock subclasses. We assumed that the genes corresponding to the individual signaling and metabolic pathways listed in Table 2 likely have the most biological significance with regard to differentiating the three septic shock subclasses. Accordingly, we extracted the individual genes corresponding to these pathways (307 total genes). These 307 genes were then subjected to a leave one out cross-validation procedure using the support vector machines algorithm and the Fisher's exact test method of gene selection [22]. This cross-validation procedure yielded 89 correct subclass predictions (subclass A, B, or C) out of 98 (91%).

From the 307 genes used in the cross-validation procedure, we then extracted the top 100 genes based on subclass prediction strength. These top 100 most predictive genes were then uploaded to the IPA application and the analytical output was again focused on enrichment for genes corresponding to signaling and metabolic pathways. The top five signaling pathways derived from this analysis are shown in Table 3. All of these signaling pathways are relevant to the pathobiology of septic shock and are particularly relevant to the adaptive immune system.

**Table 3:** Top five Ingenuity Pathways Analysis-derived signaling pathways corresponding to the top 100 predictor genes identified by leave one out cross-validation procedures.

**Pathway**	***P *value**	**Number of genes**
B cell receptor signaling	2.1E-27	25

T cell receptor signaling	8.0E-16	15

Glucocorticoid receptor signaling	4.3E-15	20

Natural killer cell signaling	6.8E-14	14

Peroxisome proliferator-activated receptorα/retinoid × receptor activation	4.0E-12	15

**Figure 5:**
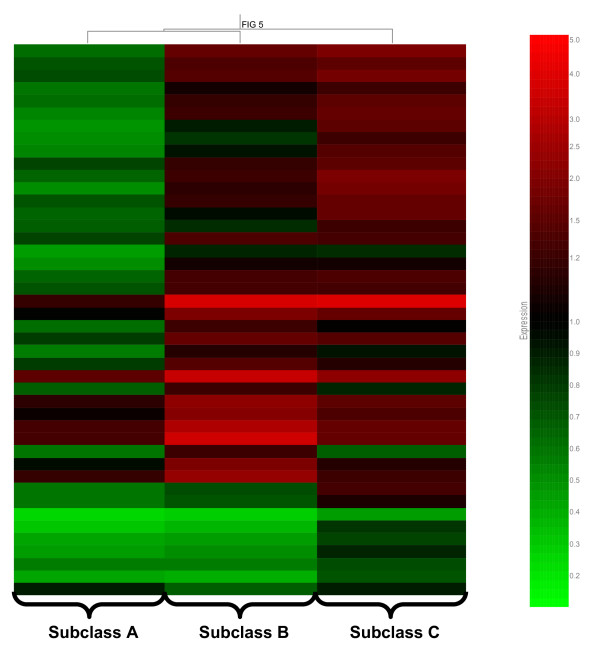
**Hierarchical clustering of the 44 genes shown in Table 4**. Each gene is colored by the median expression values for each of the respective septic shock subclasses, as labeled at the bottom of the figure.

Forty-four of the top 100 predictive genes corresponded to the top five signaling pathways shown in Table 3. These 44 genes (listed in Table 4) were subjected to hierarchical clustering based on the median expression values for each septic shock subclass, as shown in Figure 5. The gene expression pattern depicted in Figure 5 demonstrates that septic shock subclass A patients had generalized repression of these 44 genes, relative to subclasses B and C. Thus, patients in septic shock subclass A, having a clinical phenotype characterized by higher mortality, higher illness severity, and a higher degree of organ failure, are also characterized by repression of genes corresponding to key signaling pathways of the adaptive immune system. Importantly, the median absolute lymphocyte counts per mm^3 ^(IQR) were not significantly different across the three subclasses: subclass A 1,585 (788–2,854); subclass B 1,530 (601–2,947); and subclass C 2,610 (1,329–4095).

**Table 4:** Forty-four genes corresponding to the signaling pathways in Table 3.

**Affymetrix number**	**Genebank**	**Gene symbol**	**Description**
242482_at	AI682905	PRKAR1A	Protein kinase, cAMP-dependent, regulatory, type I, α
241905_at	AA579047	PIK3C2A	Phosphoinositide-3-kinase, class 2, α polypeptide
239585_at	AV735100	KAT2B	K(lysine) actetyltransferase 2B
236561_at	AV700621	TGFBR1	Transforming growth factor, β receptor I
236283_x_at	AA287921	PAK2	p21 (CDKN1A)-activated kinase 2
230337_at	AW241962	SOS1	Son of sevenless homolog 1 (*Drosophila*)
228343_at	AA805754	POU2F2	POU domain, class 2, transcription factor 2
228173_at	AA810695	GNAS	GNAS complex locus
227131_at	BG231756	MAP3K3	Mitogen-activated protein kinase kinase kinase 3
225927_at	AA541479	MAP3K1	Mitogen-activated protein kinase kinase kinase 1
224994_at	AA777512	CAMK2D	Calcium/calmodulin-dependent protein kinase II δ
224621_at	AA129773	MAPK1	Mitogen-activated protein kinase 1
221616_s_at	AF077053	TAF9B	TAF9B RNA polymerase II
219290_x_at	NM_014395	DAPP1	Dual adaptor of phosphotyrosine and 3-phosphoinositides
218806_s_at	AF118887	VAV3	vav 3 oncogene
216033_s_at	S74774	FYN	FYN oncogene related to SRC, FGR, YES
215605_at	AU145806	NCOA2	Nuclear receptor coactivator 2
214322_at	AA284757	CAMK2G	Calcium/calmodulin-dependent protein kinase II γ
214032_at	AI817942	ZAP70	Zeta-chain (TCR) associated protein kinase 70 kDa
213579_s_at	AI459462	EP300	E1A binding protein p300
211711_s_at	BC005821	PTEN	Phosphatase and tensin homolog
211583_x_at	AF031136	NCR3	Natural cytotoxicity triggering receptor 3
210992_x_at	U90939	FCGR2C	Fc fragment of IgG, low affinity IIc, receptor for (CD32)
210162_s_at	U08015	NFATC1	Nuclear factor of activated T cells, calcineurin-dependent 1
210031_at	J04132	CD247	CD247 molecule
209685_s_at	M13975	PRKCB1	Protein kinase C, beta 1
207387_s_at	NM_000167	GK	Glycerol kinase
207238_s_at	NM_002838	PTPRC	Protein tyrosine phosphatase, receptor type, C
206854_s_at	NM_003188	MAP3K7	Mitogen-activated protein kinase kinase kinase 7
205931_s_at	NM_004904	CREB5	cAMP responsive element binding protein 5
205841_at	NM_004972	JAK2	Janus kinase 2 (a protein tyrosine kinase)
205456_at	NM_000733	CD3E	CD3e molecule, epsilon (CD3-TCR complex)
204297_at	NM_002647	PIK3C3	Phosphoinositide-3-kinase, class 3
203837_at	NM_005923	MAP3K5	Mitogen-activated protein kinase kinase kinase 5
203561_at	NM_021642	FCGR2A	Fc fragment of IgG, low affinity IIa, receptor (CD32)
203266_s_at	NM_003010	MAP2K4	Mitogen-activated protein kinase kinase 4
203140_at	NM_001706	BCL6	B cell CLL/lymphoma 6 (zinc finger protein 51)
202789_at	AL022394	PLCG1	Phospholipase C, gamma 1
202625_at	AI356412	LYN	v-yes-1 Yamaguchi sarcoma viral oncogene homolog
1568943_at	BC027960	INPP5D	Inositol polyphosphate-5-phosphatase, 145 kDa
1565703_at	AL832789	SMAD4	SMAD, mothers against DPP homolog 4 (*Drosophila*)
1558732_at	AK074900	MAP4K4	Mitogen-actvated protein kinase kinase kinase kinase 4
1558135_at	BQ709323	TAF11	TAF11 RNA polymerase II
1557675_at	BI496583	RAF1	V-raf-1 murine leukemia viral oncogene homolog 1

### Regulation of zinc biology-related genes across the septic shock subclasses

**Figure 6:**
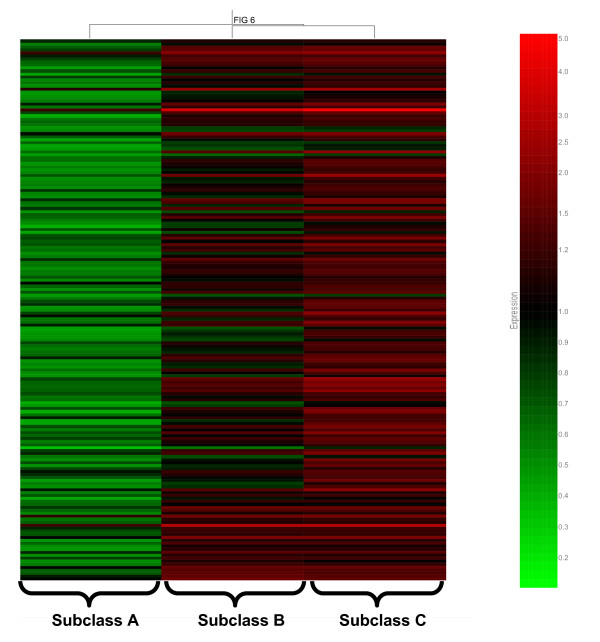
**Hierarchical clustering of the 181 genes corresponding to zinc biology-related functional annotations and derived from K-means cluster 8 shown in Figure 4**. Each gene is colored by the median expression values for each of the respective septic shock subclasses, as labeled at the bottom of the figure.

Our previous studies demonstrated that pediatric septic shock is broadly characterized by large-scale repression of genes that either depend on normal zinc homeostasis for normal function or directly participate in zinc homeostasis [9,11-13]. In the current analysis, we determined if zinc biology-related genes were differentially regulated across the three septic shock subclasses. The 10 K-means clusters depicted in Figure 4 were interrogated for enrichment of zinc biology-related annotations ('zinc', 'zinc finger', 'zinc ion binding', 'metal binding', and 'metal ion binding') by uploading the individual gene cluster lists to the DAVID database. Cluster 8 was most significantly enriched for these functional annotations with *P *values ranging from 2.3E-5 to 9.7E-10 (data not shown). The genes corresponding to these zinc biology-related functional annotations were identified (181 genes) and subjected to hierarchical clustering based on the median expression values for each septic shock subclass, as shown in Figure 6. The gene expression pattern depicted in Figure 6 demonstrates that septic shock subclass A patients had generalized repression of these 181 zinc biology-related genes, relative to subclasses B and C. Thus, our previous observations regarding large-scale repression of zinc biology-related genes appears to be a relatively unique feature of patients in septic shock subclass A.

## Discussion

Multiple clinical trials have been conducted in patients with septic shock and most have been based on strategies targeting various components of the immune or inflammatory system [23]. Despite being well supported by quality preclinical data, the majority of these strategies have failed when tested by way of randomized, placebo-controlled trials. One major reason for these recurrent failures is the broad heterogeneity intrinsic to the syndrome of septic shock [1]. That is, it is unlikely that any single immune or inflammatory modulating therapy will be beneficial to a heterogeneous group of patients with septic shock. Thus, identification of septic shock subclasses could facilitate the design of more specifically targeted clinical trials having a higher likelihood of demonstrating efficacy.

Herein we have attempted to discover and identify pediatric septic shock subclasses by leveraging the discovery potential of high throughput genomics, a strategy that is now well established in the field of cancer [24-29]. The foundation of our data is a discovery-oriented expression filter, which identified genes having at least two-fold expression difference in between 25% to 50% of the septic shock patients, relative to the median of controls. While this strategy is certainly not the only valid approach to the goals of our study, it allowed us to initially identify three putative subclasses of patients with septic shock based on unsupervised hierarchical clustering. The ability of this strategy to effectively identify subgroups is well demonstrated by the results of direct statistical testing, which mandated the use of a correction for multiple comparisons procedure (Bonferroni) generally thought to be overly stringent for microarray data [30,31]. The large number of differentially regulated genes identified by stringent, direct statistical testing strongly suggests that the three putative septic shock subclasses are biologically plausible.

The differentially regulated genes identified by direct statistical testing were able to distinguish three broad septic shock subclasses when subjected to hierarchical clustering and principal component analysis, thus further supporting the assertion of biological plausibility. K-means clustering of these differentially regulated genes generated coordinately regulated gene clusters corresponding to multiple signaling and metabolic pathways relevant to the pathobiology of septic shock. Importantly, the cluster maps derived from K-means clustering demonstrate that these signaling and metabolic pathways are differentially regulated across the three subclasses, thus illustrating, at a genomic level, the concept that any one specific immune or inflammatory modulating therapeutic strategy will not be applicable to a heterogeneous cohort of patients with septic shock.

The three septic shock subclasses identified by expression profiling differed significantly with respect to important clinical phenotypes. Specifically, patients in subclass A had a higher level of illness severity, a higher degree of organ failure, and a higher mortality rate compared with the other two subclasses. In addition, patients in subclass A were younger than patients in subclass B. The largest epidemiologic study of pediatric septic shock to date, by Watson *et al*. [32], demonstrated that children between the ages of 1 and 12 months had the highest mortality rate (13.5%), when compared with other age groups. Thus, the demonstration that the subclass having the highest mortality rate is composed of younger children is consistent with the existing epidemiologic literature. However, the mortality rate of subclass A (36%) is substantially higher than the overall mortality reported by Watson *et al*. [32] (10.3%), as well as the overall mortality rate (17.3%) reported in the largest pediatric septic shock interventional trial to date [33]. In addition, the median ages between patients in subclass A and C were not significantly different. Thus, it is likely that the higher mortality rate in subclass A is, at least in part, a direct manifestation of the gene expression profile that identified the subclass, rather than a simple artifact of having identified a subclass that was significantly younger.

Based on leave one out cross-validation procedures and subsequent extraction of the top 100 predictor genes, the most distinguishing gene expression signature of the subclass A patients was repression of genes corresponding to the adaptive immune system. This pattern of gene repression does not appear to be an artifact of lymphopenia, in as much as the absolute lymphocyte counts were not significantly different across the three subclasses. In addition, subclass A patients were characterized by repression of genes corresponding to glucocorticoid receptor signaling, an intriguing finding given the current controversies surrounding glucocorticoid treatment in septic shock [34]. Finally, our subanalysis focused on repression of genes having zinc biology-related functional annotations demonstrated that repression of zinc biology-related genes was also a distinguishing feature of the subclass A patients. Thus, we conclude that subclass A patients are particularly distinguished from subclass B and C patients by gene repression patterns corresponding to adaptive immunity, glucocorticoid receptor signaling, and zinc-related biology. This pattern of gene repression correlates with higher illness severity, a greater degree of organ failure, and higher mortality in subclass A patients.

Since our data are based on whole blood-derived RNA, it is possible that some of the gene expression patterns that distinguish the three subclasses are a reflection of different distributions of white blood cell populations, rather than within-cell differences in gene expression. As stated above, however, the absolute lymphocyte counts were not significantly different across the three subclasses, thus suggesting that our data are not simply artifacts of differential white blood cell populations. Nonetheless, we are currently in the process of directly addressing this important question by generating expression data from leukocyte subset-specific RNA.

The existing literature supports the biological plausibility of our data at two broad levels. First, our conceptual framework of the pathobiology of septic shock has evolved over the last decade to include the concept of immune paralysis [35-40]. Whereas septic shock has been traditionally viewed as being a reflection of uncontrolled hyper inflammation (an innate immunity problem), it is now thought that septic shock also has a strong, perhaps predominant, anti-inflammatory component that can be manifest as immune suppression and the relative inability to effectively clear an infectious challenge (an adaptive immunity problem). The finding that subclass A patients are characterized by repression of key adaptive immunity genes is well in line with this concept.

Second, normal zinc homeostasis seems to be absolutely critical for normal functioning of both the innate and adaptive immune systems [41,42]. Thus, we have postulated that abnormal zinc homeostasis may be linked to adaptive immune dysfunction in children with septic shock [10]. In support of this assertion, we previously demonstrated that non-survivors of pediatric septic shock had abnormally low serum zinc concentrations compared with survivors [11]. Potential links between altered zinc homeostasis, adaptive immune function, and septic shock are the subject of ongoing work in our basic and translational research programs.

## Conclusion

We have demonstrated the existence of three broad subclasses of children with septic shock based on gene expression profiling conducted within the first 24 hours of admission to the intensive care unit with septic shock. Broadly, the three subclasses demonstrate differential regulation of genes corresponding to multiple signaling and metabolic pathways relevant to the pathobiology of septic shock, thereby illustrating the important concepts of patient heterogeneity at a genomic level and the need to design more specifically targeted therapies. On a more specific level, the three subclasses are characterized by differential regulation of genes corresponding to the adaptive immune system and zinc-related biology, and these patterns of gene regulation correlate with distinct and relevant clinical phenotypes. Genome-level subclassification of septic shock may one day allow for the design of more specifically targeted therapies, and our data indicate that the adaptive immune system and zinc homeostasis may be appropriate targets to explore further.

## Abbreviations

ANOVA: analysis of variance; DAVID: database for annotation, visualization, and integrated discovery; ERK: epidermal growth factor; GEO: gene expression omnibus; IPA: ingenuity pathways analysis; IQR: intra-quartile range; JAK: Janus kinase; MAPK: mitogen-activated protein kinase; MIAME: minimum information about a microarray experiment; NF-κB: nuclear factor kappa-light-chain-enhancer of activated B cells; PPARα: peroxisome proliferator-activated receptor; PRISM: pediatric risk of mortality; RMA: robust multiple-array average; RXRα: retinoid × receptor; STAT: signal transducers and activator of transcription.

## Competing interests

The authors declare that they have no competing interests.

## Authors' contributions

HRW directed the overall study design and analysis, and drafted the manuscript. NC, RL, GLA, NJT, DFW, RJF, NA, KM, PAC, and TPS contributed clinical data and biological samples to the database. MM supervised the clinical database and all administrative aspects of the study. KO supervised the processing of all biological samples. TPS collaborated with HRW in the initial database development. All authors reviewed the manuscript prior to submission.

## Pre-publication history

The pre-publication history for this paper can be accessed here:


